# Stability Indicating RP-HPLC Method for the Simultaneous Determination of Atorvastatin Calcium, Metformin Hydrochloride, and Glimepiride in Bulk and Combined Tablet Dosage Form

**DOI:** 10.1155/2014/754695

**Published:** 2014-10-29

**Authors:** Devi Ramesh, Mohammad Habibuddin

**Affiliations:** ^1^Government Polytechnic for Women, Gujarathipeta, Srikakulam, Andhra Pradesh 532005, India; ^2^Adept Pharma & Bioscience Excellence Private Limited, 10-3-561/3A/102, Vijayanagar colony, Hyderabad, Andhra Pradesh 500057, India

## Abstract

A simple, rapid, and precise RP-HPLC method for simultaneous analysis of atorvastatin calcium, metformin hydrochloride, and glimepiride in bulk and its pharmaceutical formulations has been developed and validated. These drugs were separated by using Grace Smart Altima C-8 column (250 × 4.6 mm, 5-*μ*m) with a mobile phase consisting of acetonitrile : phosphate buffer (60 : 40 (v/v), pH 3.0) at a flow rate of 1 mL/min, injection volume 25 *µ*L, and detection at 235 nm. Metformin, atorvastatin, and glimepiride were eluted with retention times of 2.57 min, 7.06 min, and 9.39 min, respectively. The method was validated for accuracy, precision, linearity, specificity, and sensitivity in accordance with ICH (Q2B) guidelines. The results of all the validation parameters were found to be within the acceptable limits. The calibration plots were linear over the concentration ranges from 10 to 150 *µ*g/mL, 20 to 200 *µ*g/mL, and 10 to 150 *µ*g/mL for atorvastatin, metformin, and glimepiride, respectively. The accuracy and precision were found to be between 98.2%–105% and ≤2% for three drugs. Developed method was successfully applied for the determination of the drugs in tablet dosage form and recovery was found to be >98% for three drugs. The degradation products produced as a result of stress studies did not interfere with drug peaks.

## 1. Introduction

**Figure 1:**
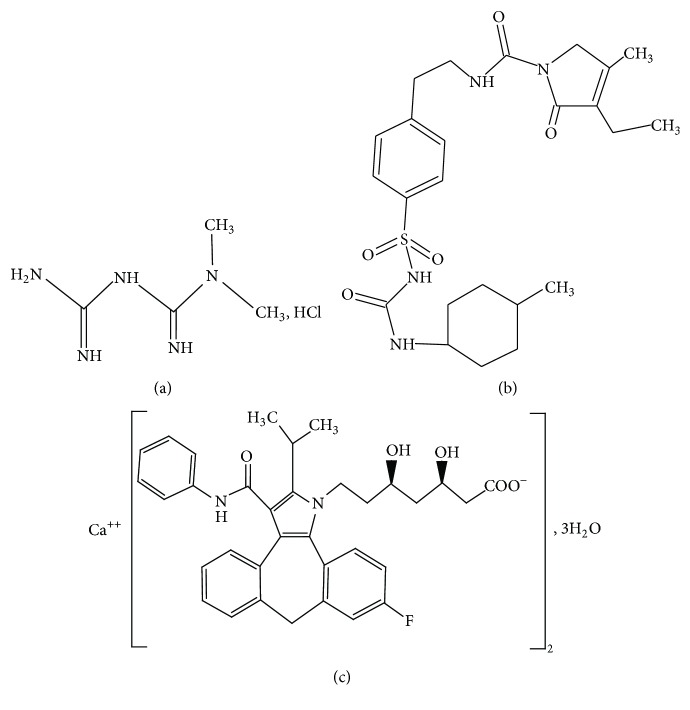
Chemical structures of drugs used in this method. (a) Metformin hydrochloride, (b) glimepiride, and (c) atorvastatin calcium.

Metformin hydrochloride (MET) is an oral biguanide and chemically it is N,N-dimethylimidodicarbonimidic diamide (Figure 1(a)), which reduces the elevated blood glucose concentration in patients with diabetes but does not increase insulin secretion. It does not lower the glucose levels in nondiabetic subjects [1, 2]. Chemically glimepiride (GLM) is (Figure 1(b)) 3-ethyl-4-methyl-N-[2-[4-[(4-methylcyclohexyl) carbamoylsulfamoyl]phenyl]ethyl]-5-oxo-2H-pyrrole-1-carboxamide. GLM is an oral hypoglycemic agent that acts by stimulating the release of insulin from functioning pancreatic beta cells and increasing sensitivity of peripheral tissues to insulin [3–5]. Chemically, atorvastatin calcium (ATR) (Figure 1(c)) is (*β*R, 8R)-2(4-flurophenyl)-*α*,*δ*-dihydroxy-5-(1-methylethyl)-3-phenyl-4-[(phenylamino)carbonyl]-1H-pyrrole-1-heptonoic acid trihydrate [6]. Atorvastatin calcium (ATR) is a competitive inhibitor of HMG-CoA reductase used in the treatment of hyperlipidemia [7].

Fixed dose combination drugs are used to maintain steady state of glucose and lipid levels in blood plasma. Tablet dosage form (Tripill) contains an oral lipid lowering agent, atorvastatin, and two oral antihyperglycemic drugs, glimepiride and metformin hydrochloride. The combination of these three drugs complements each other and provides a reduction in plasma cholesterol along with glycemic control, thereby providing comprehensive control of diabetes associated with dyslipidemia.

The objective of the present investigation is to develop a simple and novel method for the simultaneous estimation MET, GLM, and ATR by employing HPLC. The literature search reveals that, few methods were reported for this combination in dosage form and in plasma [8–10], there are some other methods that are reported for individuals as well as individual drugs with other drug combinations [11–20]. The analytical method employed for the quantitative determination of drug in formulation plays a significant role in the evaluation and interpretation of drug release from the formulation. Therefore, a complete validation of analytical methods was performed according to ICH guidelines [21] to yield reliable results that could be satisfactorily interpreted. So, the proposed method can be employed for the determination of three dugs in bulk and combination formulations (Tripill) as well as in future studies of these drugs.

## 2. Materials and Methods 

### 2.1. Instrumentation

The instruments employed in this study are HPLC-Perkin Elmer, UV-200 series with the total chrome Software, Waltham, USA; sonicator-Sharp Analytical, Hyderabad, India; analytical balance-Sartorius, German; Millipore Direct-Q 3 U.V. USA; pH meter-Systronics, Ahmadabad, India.

### 2.2. Standards and Chemicals

ATR, MET, and GLM were gift samples obtained from Aurobindo Pharma (Hyderabad, India). Purified water was obtained from a Millipore Direct-Q 3 U.V. Acetonitrile of HPLC grade, o-phosphoric acid, and sodium dihydrogen phosphate were of A.R. grade and were purchased from Merck, Pvt. Ltd (Mumbai, India). Tripll 2 tablet dosage form with concentrations of ATR-10 mg, MET-500 mg, and GLM-2 mg was purchased from the market.

### 2.3. Stock and Working Solution Preparation

*Preparation of Standard Stock Solution*. Accurately weighed 10 mg of each drug, that is, MET, GLM, ATR and transferred into a 10 mL volumetric flask dissolved with small amount of solvent (acetonitrile : water 50 : 50) and made up of the volume with 50 : 50 v/v water and acetonitrile. Daily working standard solutions of mixture were prepared by suitable dilution of the stock solution with the mobile phase. 

*Preparation of a Buffer*. Accurately weighed 2.72 g of potassium dihydrogen orthophosphate dissolved in 1000 mL of HPLC grade water and pH was adjusted to 3.0 by using orthophosphoric acid.

### 2.4. Chromatographic Conditions

The chromatographic separation was done by using Grace Smart Altima C-8 column (250 × 4.6 mm, 5 *μ*) with mobile phase acetonitrile: phosphate buffer pH 3.0 (60 : 40% v/v) at a flow rate of 1 mL/min and detection wavelength was 235 nm with 25 *μ*L of injection volume.

### 2.5. Method Optimization

The method development, top priority was given for the complete separation of drugs. The chromatographic method was optimized by changing various parameters, such as pH of the mobile phase, organic solvent and buffer used in the mobile phase, and composition of the mobile phase on trial error basis by varying one parameter and keeping all other conditions constant.

### 2.6. Method Validation

The validation parameters like linearity, sensitivity, accuracy, precision, recovery, and stability of drugs were studied according to the ICH guidelines [21].

#### 2.6.1. Selectivity

Selectivity was studied by comparing the chromatograms obtained from the blank sample with the chromatogram obtained from a standard drug mixture.

#### 2.6.2. Linearity

The linearity of this method was evaluated by linear regression analysis, using least square method, and the linearity of drugs was found in the concentration range of 20–200 *μ*g/mL for MET, 10–150 *μ*g/mL for ATR and GLM. Calibration standards are prepared by spiking the required volume of working standard (200 *μ*g/mL) solution into different 10 mL volumetric flasks and volume made up with mobile phase to yield concentrations of 10, 20, 30, 40, 50, 100, 150, and 200 *μ*g/mL of MET, GLM, and ATR. The resultant peak area of each drug was measured. Calibration curve is plotted between peak areas of drug against concentration of the drug.

#### 2.6.3. Sensitivity

The LOD and LOQ of this method were verified based on the standard deviation of response, slope.

#### 2.6.4. Intraday and Interday Precision and Accuracy and Recovery

Intra- and interday accuracy and precision of this method were determined at three different concentration levels in 3 different days. On each day, three replicates were analyzed with independently prepared calibration curves. The accuracy and precision were expressed as percentage accuracy and relative standard deviation (RSD), respectively.

#### 2.6.5. Recovery

The recovery study was carried out at three levels of 80% (40 *μ*g/mL), 100% (50 *μ*g/mL), and 120% (60 *µ*g/mL) of standard drug was added to the extracted solution of formulation, diluted the solution and injected into HPLC, then calculated the recovery.

#### 2.6.6. Robustness

Robustness of the method was done by changing slight variation in the parameters like mobile phase composition, flow rate, and wavelength. Present method did not show any significant change when the critical parameters were modified (i.e., mobile phase composition, flow rate, and pH of buffer).

#### 2.6.7. Solution Stability

The stability of the drug solution was determined for the short-term stability and autosampler stability.* Short-term stability* was carried out by keeping at room temperature (25°C) for 24 h.* Autosampler stability* was determined by storing the samples for 24 h in the autosampler. Each sample injected three times into HPLC and concentrations obtained were compared with the nominal values of the quality control (QC) samples.

#### 2.6.8. Forced Degradation Study

The stress studies were carried out by taking 100 mg of each drug into 100 mL volumetric flask and added 5 mL of 1 M hydrochloric acid for acid degradation, 5 mL of 1 M sodium hydroxide for alkali degradation, and 10 mL of water for hydrolytic degradation; then samples were kept in a water bath at 60°C for 1 h. After heating, the solutions in volumetric flasks were neutralized, that is, 1 M HCl with 1 M NaOH, 1 M NaOH with 1 M HCl and volume made up with the mobile phase separately. Photodegradation was carried out by the drug was kept under UV light 254 nm for 24 h. Then diluted the drug solution with mobile phase to get suitable concentration within the linearity range and injected the samples into the HPLC.

### 2.7. Analysis of Marketed Formulation

20 tablets were weighed and finely powdered, and an accurately weighed sample of powdered tablets equivalent to 10 mg of ATR, 500 mg of MET, and 2 mg of GLM (equivalent to one tablet) was extracted with different extraction solvents like acetonitrile, methanol, water, and mobile phase. The recovery of drugs (ATR and GLM) was found to be less than 50% when extracting by using 100% water and the recovery of MET was found to be less than 90% when extraction carried out by using acetonitrile. The 100% recoveries of all three drugs were found when extraction carried out by using the combination of water and acetonitrile (50 : 50) as extraction solvent. Hence, the composition of 50 : 50 v/v of acetonitrile: water was used as extraction solution. The powder equivalent to one tablet was transferred and extracted with 50 : 50 of acetonitrile : water in a 100 mL volumetric flask and sonicated for 15 min. This solution was filtered through Whatman number 1 filter paper. The solution obtained was diluted with the mobile phase so as to obtain a concentration in the range of linearity previously determined, and then filtered through 0.22 *µ* syringe filter. The amount of drugs recovered was calculated from the respective linear graph.

## 3. Results and Discussion

During the method development, top priority was given for the complete separation of drugs. The chromatographic method was optimized by changing various parameters, such as pH of the mobile phase, organic solvent and buffer used in the mobile phase, and composition of the mobile phase on trial error basis. Phosphate buffer in various strengths are tried along with methanol and acetonitrile as organic solvent. A mixture of acetonitrile and phosphate buffer with different pH values was tried. At pH 3.0 the separation was good enough; then the proportions of acetonitrile and phosphate buffer pH 3.0 were tested as a mobile phase with Grace Smart C-8 columns. The mobile phase composition of 40 : 60 v/v phosphate buffer : acetonitrile was shown to have good resolution and retention time with minimal tailing factor in acceptable range. The method was optimized with the mobile phase composition of acetonitrile and phosphate buffer 60 : 40 (v/v). Buffer molarity of 10, 20, and 50 mM was tested. There were no significant changes in the chromatographic response and peak shape with change in buffer molarity. A buffer molarity of 20 mM was selected for further analysis.

**Figure 2:**
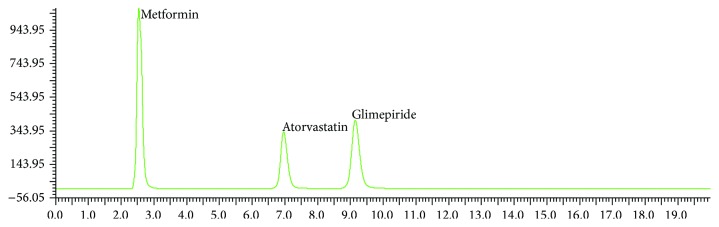
Standard chromatogram of MET, GLM, and ATR.

After several trials, the method was optimized as a mixture of 20 mM potassium dihydrogen phosphate buffer (pH 3.0) and acetonitrile (40 : 60 v/v) at a flow rate of 1 mL/min and at 235 nm by using Grace Smart, Altima C-8 column. These chromatographic conditions achieved satisfactory resolution, retention time, and tailing for three drugs of MET, GLM, and ATR.  Figure 2 shows that chromatogram of standard drug mixture and these are well separated from each other.

The standard mixture solution was used as a system suitability solution it was injected into HPLC. The retention time, tailing factor, resolution, and theoretical plates for each drug were observed. The percentage relative standard deviation (%RSD) of five consecutive injections for each parameter was calculated. The system suitability parameters of the present method were found to be within acceptable limits. The system suitability data are presented in Table 1. The acceptable limits of the resolution between two adjacent peaks should be ≥2 and tailing factor should be ≤2 [22] and the %RSD of these values should be ≤2. System suitability tests confirmed that the chromatographic system was adequate for the analysis planned to be done.

The linearity was performed and calibration curve is plotted between peak areas of drug against concentration of the drug. The curve was linear over the range of 20–200 *μ*g/mL for MET and 10–150 *μ*g/mL for ATR and GLM. The regression equations of three drugs were *y* = 79069*x* − 23231  (*r* ^2^ = 0.998) for MET, *y* = 33694*x* − 45799  (*r* ^2^ = 0.998) for ATR, and *y* = 47641*x* − 49907  (*r* ^2^ = 0.999) for GLM. The results of intra- and interday precision was shown in Table 2. The %RSD was found to be less than 2 for all the drugs which indicates that the method is precise. Recovery experiments were done to determine the accuracy of method. The results are represented in Table 3. The data indicated good accuracy and reproducibility.

Present method did not show any significant change when the critical parameters were modified. The tailing factor for the drugs was always less than 2.0 and the components were well separated under all the changes carried out (i.e., mobile phase composition, flow rate, and pH of buffer). Considering the modifications in the system suitability parameters and the specificity of the method, as well as carrying the experiment at room temperature, may indicate that the proposed method was robust.

The stability of the drug was studied for short-term and autosampler stability using the QC samples. The samples were analyzed and compared with freshly analyzed QC samples; no differences were found in accuracy and precision. The stability data presented in Tables 4 and 5 indicate that there were no major changes observed in this study.

**Figure 3:**
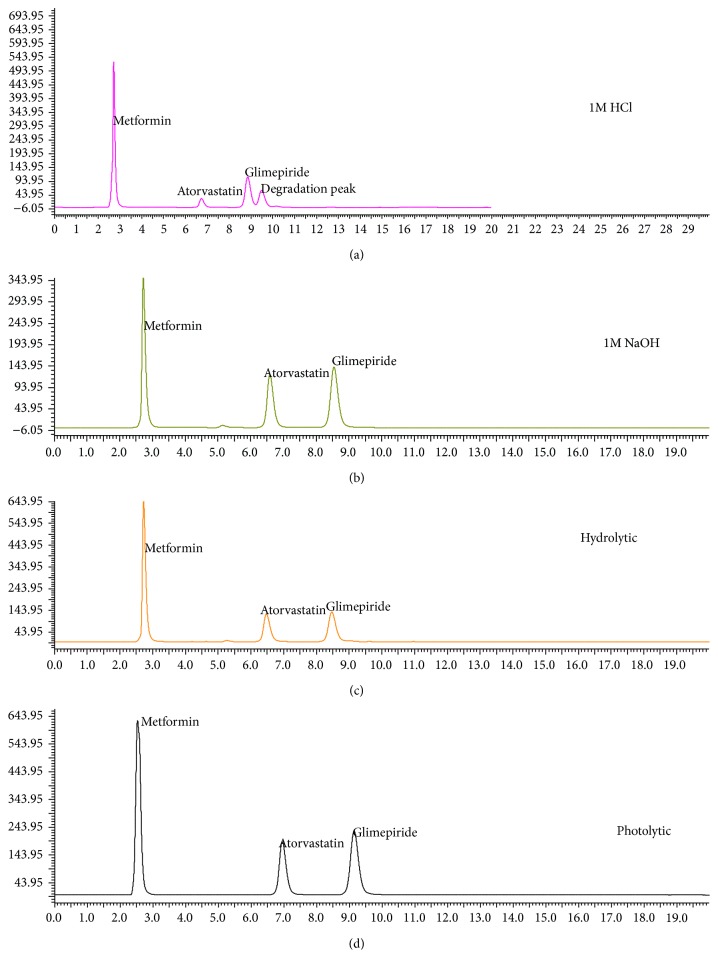
Chromatograms of MET, GLM, and ATR under stress conditions (a) 1 M hydrochloric acid, (b) 1 M sodium hydroxide, (c) neutral (hydrolysis), and (d) photolytic.

Forced degradation studies were carried out in acid, base, and neutral conditions; ATR was degraded more (30.19%) in acidic conditions than basic and neutral conditions. In basic conditions MET was degraded more (30.5%) compared to the other two drugs; no degradation was found in hydrolytic conditions. The amount of GLM in acid and hydrolytic conditions was reduced, but there was no reduction in the amount of GLM in basic conditions. The results of stress study indicate that ATR is unstable under these conditions. Two degradation peaks were appearing at retention time of 5.2 min and 9.62 min in the chromatogram under applied stress studies. These degradation peaks were not interfering with their parent peaks. Hence this method could be employed for the determination of these three drugs, that is, MET, GLM, and ATR in the presence of their degradation product. Under photodegradation study ATR degraded 4% and no degradation was found for MET and GLM. The chromatograms of stress conditions are shown in Figure 3 and the percentage degradation of each in stress studies of MET, GLM, and ATR were represented in (Table 6).

**Figure 4:**
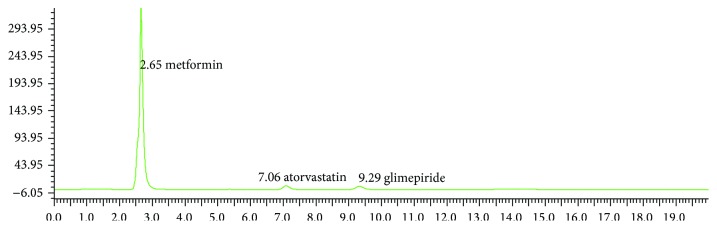
Chromatogram of ATR, MET, and GLM from the formulation.

The results of assay of dosage form percentage recovery was found to be more than 98% for all the drugs (ATR, MET, and GLM); the data was represented in Table 7; the chromatogram from formulation was shown in (Figure 4).

## 4. Conclusions

The developed method possesses good selectivity and specificity; there is no interference found in the blanks at retention times of ATR, MET, and GLM and good correlation between the peak area and concentration of the drug under optimized conditions. The recovery studies are found to be >98% for three drugs. The observation of %RSD less than 2 for both intra- and interday measurements indicates a high degree of precision. In the present method, a Grace Smart, Altima C-8 column has been used at a flow rate of 1 mL/min. The method was optimized with low injection volume. The stability of ATR, MET, and GLM were found to be within the limits indicating that there is no degradation of drugs during the daily analysis. This method was applied for the simultaneous determination of ATR, MET, and GLM in tablet dosage form.

## Figures and Tables

**Table 1:** System suitability parameters of ATR, MET, and GLM.

Parameter	MET		ATR		GLM		Specifications [22]
	Mean ± SD	%RSD	Mean ± SD	%RSD	Mean ± SD	%RSD	
Rt	2.57 ± 0.04	1.75	7.06 ± 0.13	1.90	9.39 ± 0.11	1.23	RSD ≤ 2
TF	1.25 ± 0.01	0.92	1.15 ± 0.01	1.00	1.11 ± 0.01	0.89	TF ≤ 2
Rs	9.38 ± 0.18	1.92	—	—	5.27 ± 0.06	1.18	
TP	3556 ± 64	1.82	4872 ± 93	1.92	6280 ± 30	0.489	*N* > 2000

Rt; retention time, TF: tailing factor, Rs: resolution, and TP: theoretical plates; *n* = 5.

**Table 2:** Intra and Interday accuracy and precision of ATR, MET, and GLM.

Conc. in *µ*g/mL	MET		ATR		GLM	
	Mean ± SD	%RSD	Mean ± SD	%RSD	Mean ± SD	%RSD
Intraday (*n* = 6)						
10	—	—	99.82 ± 1.151	1.153	105.02 ± 0.236	0.225
20	99.36 ± 0.137	0.137	106.73 ± 1.567	1.468	104.27 ± 0.835	0.801
50	100.08 ± 1.994	1.992	98.95 ± 0.222	0.225	100.78 ± 0.303	0.301
100	102.67 ± 0.393	0.383	102.04 ± 0.204	0.200	103.14 ± 0.610	0.591

Interday (*n* = 9)						
10	—	—	99.15 ± 0.593	0.598	103.57 ± 1.422	1.373
20	100.43 ± 1.672	1.665	103.17 ± 1.328	1.287	102.45 ± 0.355	0.346
50	98.20 ± 1.007	1.025	100.22 ± 0.695	0.694	100.82 ± 0.217	0.215
100	101.45 ± 0.207	0.204	98.37 ± 1.338	1.360	100.92 ± 1.082	1.072

**Table 3:** Recovery study of ATR, MET, and GLM.

Add. conc.	MET		ATR		GLM	
	Obt. conc.	(%) Recovery	Obt. conc.	(%) Recovery	Obt. conc.	(%) Recovery
80% (40)	39.49 ± 0.12	98.74 ± 0.30	40.02 ± 0.25	100.05 ± 0.64	40.57 ± 0.36	101.44 ± 0.92
100% (50)	50.49 ± 0.33	100.98 ± 0.67	48.72 ± 0.17	97.45 ± 0.35	49.05 ± 0.03	98.1 ± 0.07
120% (60)	59.8 ± 0.70	99.67 ± 1.17	61.17 ± 0.93	101.96 ± 1.55	60.51 ± 0.16	100.86 ± 0.27

Add. conc.: added concentration; Obt. conc.: obtained concentration; *n* = 3.

**Table 4:** Short-term stability data of ATR, MET, and GLM.

Conc. in *µ*g/mL	MET		ATR		GLM	
	Mean ± SD	%RSD	Mean ± SD	%RSD	Mean ± SD	%RSD
10	—		97.17 ± 1.101	1.133	102.95 ± 1.046	1.016
20	99.66 ± 0.337	0.338	100.15 ± 1.813	1.793	101.69 ± 0.644	0.634
50	100.5 ± 1.108	1.055	99.49 ± 0.313	0.314	100.79 ± 0.414	0.411
100	101.43 ± 0.199	0.197	99.56 ± 1.277	1.283	102.43 ± 1.079	1.054

**Table 5:** Autosampler stability data of ATR, MET, and GLM.

Conc. in *µ*g/mL	MET		ATR		GLM	
	Mean ± SD	%RSD	Mean ± SD	%RSD	Mean ± SD	%RSD
10	—		100.38 ± 0.942	0.938	99.69 ± 1.05	1.054
20	101.29 ± 1.089	1.075	100.36 ± 0.71	0.707	100.86 ± 0.191	0.189
50	98.82 ± 0.649	0.657	98.56 ± 0.622	0.631	101.13 ± 0.237	0.234
100	99.94 ± 1.478	1.479	99.04 ± 0.667	0.674	100.27 ± 1.043	1.04

**Table 6:** Forced degradation studies data of ATR, MET, and GLM.

	MET		ATR		GLM	
	%assay	%Drdn.	%assay	%Drdn.	%assay	%Drdn.
Acid	99.52	0.48	69.8	30.19	93.6	6.4
Base	69.5	30.5	88.5	11.49	100.2	—
Hydrolysis	101.46	—	92.91	7.08	91.14	8.86
Photolysis	99.82	0.18	96.2	3.8	99.25	0.75

Drdn: degradation; *n* = 3.

**Table 7:** Assay of formulation.

	MET (500 mg)		ATR (10 mg)		GLM (2 mg)	
	Amount	Assay (%)	Amount	Assay (%)	Amount	Assay (%)
Tripill 2	508.54 ± 13.61	101.70 ± 2.72	9.88 ± 0.097	98.89 ± 0.97	1.96 ± 0.021	98.18 ± 1.06

*n* = 5. Amount mentioned in brackets are label claim of tablet.

## References

[1] Hermann L. S., Kulhmann I., Plus W. (1995). Clinical pharmacology of biguanides. *Hand Book of Experimental Pharmacology*.

[2] http://www.drugbank.ca/drugs/DB00331.

[3] http://www.drugbank.ca/drugs/DB00222.

[4] Nissen S. E., Nicholls S. J., Wolski K. (2008). Comparison of pioglitazone vs glimepiride on progression of coronary atherosclerosis in patients with type 2 diabetes: the PERISCOPE randomized controlled trial. *JAMA*.

[5] Davis S. N., Brunton L. L., Lazo J. S., Parker K. L. (2005). 60. Insulin, oral hypoglycemic agents, and the pharmacology of the endocrine pancreas. *Goodman & Gilman's The Pharmacological Basis of Therapeutics*.

[6] (2007). *Indian Pharmacoepoea*.

[7] Malinowski J. M. (1998). Atorvastatin: A hydroxymethylglutaryl-coenzyme A reductase inhibitor. *The American Journal of Health-System Pharmacy*.

[8] Harish Kumar Raju C., Ramalingam P., Vamshi Krishna P. V., Ramesh N., Sreeram B. (2012). Simultaneous determination of metformin hydrochloride, atorvastatin and glimepiride in tablet dosage forms by RP-HPL. *The American Journal of PharmTech Research*.

[9] Dhaneshwar S. R., Salunkhe J. V., Bhusari V. K. (2010). Validated HPTLC method for simultaneous estimation of metformin hydrochloride, atorvastatin and glimepiride in bulk drug and formulation. *Journal of Analytical and Bioanalytical Techniques*.

[10] Srinivasa Rao P., Nageshwara Rao P., Ramakrishna G., Venkateshwarlu G. (2013). Simultaneous determination of Atorvastatin, Metformin and Glimepiride in human plasma by LC-MS/MS and its application to a human pharmacokinetic study. *Journal of Pharmaceutical Analysis*.

[11] Shah D. A., Bhatt K. K., Shankar M. B., Mehta R. S., Gandhi T. R., Baldania S. L. (2006). RP-HPLC determination of atorvastatin calcium and amlodipine besylate combination in tablets. *Indian Journal of Pharmaceutical Sciences*.

[12] Salem I. I., Idrees J., Al Tamimi J. I. (2004). Determination of glimepiride in human plasma by liquid chromatography-electrospray ionization tandem mass spectrometry. *Journal of Chromatography B*.

[13] Havaldar F. H., Vairal D. L. (2010). Simultaneous estimation of glimepiride, rosiglitazone and pioglitazone hydrochloride in the pharmaceutical dosage form. *E-Journal of Chemistry*.

[14] Praveenkumar Reddy B., Boopathy D., Mathew B., Prakash M., Perumal P. (2010). Method development and validation of simultaneous determination of pioglitazone and glimepiride in pharmaceutical dosage form by RP-HPLC. *International Journal of ChemTech Research*.

[15] Lakshmi K. S., Rajesh T. T. (2011). Development and validation of RP-HPLC method for simultaneous determination of glipizide,rosiglitazone, pioglitazone, glibenclamide and glimepiride in pharmaceutical dosage forms andhuman plasma. *Journal of the Iranian Chemical Society*.

[16] Udaykumar Rao B., Pratima Nikalje A. (2010). Determination of Glipizide, Glibenclamide and Glimepiride in a tablet dosage form in the presence of Metformin hydrochloride by Ion pair- reversed phase liquid chromatographic technique. *Journal of Analytical and Bioanalytical Techniques*.

[17] Lakshmi K. S., Rajesh T., Sharma S. (2009). Simultaneous estimation of metformin and pioglitazone by reversed phase HPLC in pharmaceutical dosage forms. *International Journal Pharmacy and Pharmaceutical Sciences*.

[18] Bhavesh D., Chethan G., Butt K. M., Shivaprakash (2007). Estimation of and pharmacokinetics of metformin in human volunteers. *Indian Journal of Pharmaceutical Education and Research*.

[19] Darwish H. W., Hassan S. A., Salem M. Y., El-Zeany B. A. (2013). Development and validation of H-point standard addition method applied for the analysis of binary mixture of amlodipine and atorvastatin. *International Journal of Pharma and Bio Sciences*.

[20] Malleswararao C. S. N., Suryanarayana M. V., Mukkanti K. (2012). Simultaneous determination of Sitagliptin phosphate monohydrate and Metformin hydrochloride in tablets by a validated UPLC method. *Scientia Pharmaceutica*.

[21] ICH Harmonized tripartite guideline, validation of analytical procedure: methodology, IFPMA.

